# Apathy and Anhedonia in Parkinson's Disease

**DOI:** 10.5402/2011/219427

**Published:** 2011-08-18

**Authors:** Yoshiaki Kaji, Koichi Hirata

**Affiliations:** Department of Neurology, Dokkyo Medical University, 880-Kitakobayashi, Mibu, Tochigi 321-0293, Japan

## Abstract

Depression, apathy, and anhedonia are often comorbid in patients with Parkinson's disease. Since the morbid states of apathy and anhedonia are complicated, these symptoms are often difficult to diagnose. Several therapeutic methods for apathy and anhedonia are considered effective. However, the validity of these methods has not been established. Similar to depression, apathy and anhedonia clearly affect the quality of life of patients and their families. Therefore, accurate diagnoses of morbid states in the early stage of the disease and corresponding appropriate treatments should be given high priority.

## 1. Introduction

Parkinson's disease (PD) is a progressive degenerative disease related to the extrapyramidal system. Patients with this disease show four major symptoms: resting tremor, muscle rigidity, akinesia, and postural reflex disturbance, all of which show a gradual progression. The prevalence rate of PD is 300 cases per 100,000. Although PD is observed over a wide age range, its crisis and prevalence rates generally increase with age. In Japan, the number of PD patients is increasing with the aging of the society. Although the primary cause of PD is the degeneration of the dopamine secretory cells in the mesencephalon substantia nigra pars compacta, the other major causative factors include the degeneration and disappearance of neurons in the noradrenaline system (locus coeruleus), the serotonin system (dorsal raphe nucleus), and the acetylcholine system (basal ganglion of Meynert). As a result, PD patients show various disorders in the motor and nonmotor systems. There has been increasing interest in PD nonmotor symptoms (NMSs). NMSs include various disorders such as mood disorders, cognitive dysfunction, mental symptoms, autonomic nervous system disorders, and perception disorders. These NMSs can be evident in the progressive stage, as well as in the early stages of functional nonmotor disorders related to cognitive, emotional, and behavioral aspects. Therefore, early detection and diagnosis of NMS are important for preventive therapies [1]. Regarding mood disorders presenting as NMS, depression [2], apathy [3], and anhedonia [4] complicate PD and are present at high rates in PD patients (approximately 40%), although these rates differ among the various reports. These complications are important factors that detrimentally affect the patients' quality of life and care burden [5]. Although apathy and anhedonia are often confused with depression, they differ from depression in terms of mechanisms, therapeutic approaches, and prognoses. If therapy is desultorily performed due to misdiagnosis, recovery from the primary disease may be delayed, activities of daily living may decrease, and the morbid states may worsen. Therefore, correct differential diagnosis is essential.

In the present study we describe the results obtained from our assessments of apathy and anhedonia occurring along with PD.

## 2. Definition and Morbid State of Apathy

### 2.1. The Relationship of Apathy with Depression

Depression is diagnosed on the basis of the diagnostic criteria defined in the Diagnostic and Statistical Manual of Mental Disorders-Fourth Edition (DSM-IV) (Table 1) [6]. Apathy is a state in which only decreased willingness is noticeable, while depressed mood and a feeling of tragedy, which are characteristic of depression, are not observed, and emotional bias is not recognized [7]. Therefore, apathy is not defined in the DSM-IV (if apathy is forcibly expressed, it can be considered as a state in which only the first half of “loss of interest or loss of pleasure” in Table 1, that is, “loss of interest,” exists). Among general physical diseases, PD is widely recognized as a disease that is easily complicated with depression. James Parkinson, who first reported PD in his “Essay on shaking palsy,” observed that it is often complicated by depression in addition to motor symptoms [8]. 

There are varying forms of depression in PD, and these forms show widely varying rates of occurrence.Reijnderset al. (2008) [9] analyzed 104 papers and reported that the least and greatest values of the prevalence rate of depression occurring along with PD are 2.7% and 89%, respectively. The prevalence rate of depression occurring along with PD was believed to vary because the evaluation methods, judgment criteria, and sampling methods varied in different studies [10]. However, the prevalence rate of depression occurring along with PD varied largely even in cases where thepopulation and examinationmethodswere similar to each other, as shown in Table 2. These results reflect the multiplicity of depression occurring along with PD. 

Only 2.4% (2.2%–7.6%) of the cases showing depression along with PD satisfy the criteria for major depression. This value is almost equal to the prevalence rate of major depression in the general population [11]. Therefore, depression occurring along with PD should be recognized as minor depression, or dysthymia, instead of major depression. Recently, Ehrt et al. [12] compared PD patients with depression to similarly aged depression patients without PD. All depression patients without PD were diagnosed with major depression, and 1/4 of the PD patients with depression were diagnosed with major depression. Moreover, the incidences of “depressive mood,” “feeling of guilt,” and “loss of pleasure” were significantly higher in depression patients without PD, and the incidence of “loss of concentration” was significantly higher in PD patients with depression. These results agree with previous findings which suggest that apathy is readily evident and that feelings of guilt, suicide attempts, and depressed mood are milder in PD patients with depression than in patients with endogenous major depression [13]. Thus, apathy in combination with PD explicitly expresses one of the pathological characteristics of depression in PD.

### 2.2. The Concept That Apathy and Depression Are Independent Morbid States

Starkstein, a researcher who proposed apathy as a disease, pointed out the following diagnostic criteria for apathy: A1, a decrease in goal-directed behavior; A2, a decrease in goal-directed cognition; A3, an emotional deficit associated with goal-directed behavior [3]. Based on the results obtained by studies on brain damage and cerebrovascular accidents, apathy is reported to be caused by damage to the dorsolateral prefrontal cortex, basal ganglion, orbital region, internal capsule (posterior limb), or the thalamus [14]. The frequency of occurrence of apathy is the highest when the frontal lobe is damaged. There is a 60% and higher probability of complication by apathy in patients with progressive-stage Alzheimer's disease [15, 16], although this figure varies widely among reports. 

The frequency of apathy occurrence is the secondly higher when the basal ganglion is damaged. Basal ganglia diseases, such as PD, progressive supranuclear palsy, and Huntington's chorea, are complicated by apathy in approximately 40% of the cases [7, 17]. In patients with PD, abnormalities in the orbitofrontal area, such as the cingulate gyrus, the corpus striatum, and the frontal lobe circuit (which plays an important role in profit-sharing-based reinforcement learning), and the mesocortical dopamine system result in motivational disorders such as apathy [18].

Symptoms common to apathy and depression include loss of interest, or loss of pleasure, retardation, fatigue, decreased emotional reactions, indifference, social degeneration, decreased initiation, decreased persistence, and loss of insight, and hypersomnia. Symptoms observed only in depression include depressed mood, suicidality, self-condemnation, excessive or inappropriate guilt, feelings of despair, and loss of appetite [19]. Apathy is a common phenotype of major depression. However, apathy syndrome, in which remarkable apathy is observed, is a different morbid state that occurs over an extended period. Depression is generally considered an “affective (emotional) disorder” accompanied by pathos. However, apathy is considered a “motivational disorder” and is not accompanied by affective disorders [20]. Unlike the findings in depression, emotion is flattened and no pathos is observed in apathy. 

Recently, Kirsch-Darrow et al. [21] evaluated depression and apathy in PD by using dystonia as a control. They found cases of PD and dystonia that were similarly complicated by apathy and depression. However, independent apathy development was observed at a high rate in PD patients only. Therefore, they concluded that apathy is the core feature of PD, and it can occur without depression, because apathy is a symptom that is independent of depression.

### 2.3. Diagnosis and Evaluation Methods of Apathy

Apathy evaluation methods can be roughly classified into subjective evaluation methods, which are based on self-recording systems, and objective evaluation methods, which are based on interviews (observation). The former is suitable for patients with mild to moderate apathy. The Apathy Evaluation Scale (AES) [22] and the Apathy Scale (AS) [3] can be used for subjective evaluation. AES consists of 18 items and was created by Marin et al. [22]. Starkstein et al.[3] created AS by amending AES to reduce the number of items to 14, and thereby making the scale easier to use for PD patients. Furthermore, AS is extensively used worldwide. In the English and Japanese versions, 14 and 16 cut-off points, respectively, are set [23]. Moreover, the validity of AS has already been established. AS is an evaluation scale based on a self-recording system; patients whose spontaneity is remarkably low and those with advanced dementia cannot answer the questions, which may limit the use of AS. 

In 2006, Sockeel et al. [24] created the Lille Apathy Rating Scale (LARS). Although LARS consists of 33 items, most of these items adopt the structured interview form with a choice between “Yes” or “No.” Therefore, LARS can be used to evaluate patients who are capable of conversing. In2011,Mulin et al. [25] examined the clinical validity of LARS for PD. LARS was recommended to be used with AS for PD. LARS and AS should be used depending on the purpose of the evaluation.

### 2.4. Examination Results Obtained in Our Institute—Part 1: Depression and Apathy

The prevalence rates of depression and apathy in PD and the depression characteristics were examined using multiple controls.

#### 2.4.1. Subjects

The PD group consisted of 46 PD patients (Table 3), and the three control groups consisted of 54 patients with acute-phase stroke (stroke group), 20 patients with medication-overuse headache (MOH; MOH group), and 10 patients with endogenous depression (endogenous depression group). The age and sex of the PD group patients were similar to those of the patients in the control groups. Table 3 shows the details of each group.

#### 2.4.2. Methods

The patients in each group were diagnosed using the diagnostic criteria defined in the DSM-IV. The levels of depression were categorized as major depression, minor depression, or dysthymia. The Hamilton Depression Scale, which is comprised of 17 items (HAM-D_17_) [26], was also used to assess the patients. Using the Japanese version [23] to determine the apathy score, we diagnosed the existence of apathy in each group. Based on the results, the prevalence rate of mood disorders in each group was calculated, and the complication rates of depression and apathy in each group were investigated. Subsequently, using subgroups of patients who were diagnosed with depression, a subanalysis was performed on nine low-ranked items of the 17 items in the HAM-D_17_; in this subanalysis, five-grade evaluation was performed to investigate the characteristics of mood disorders in PD. The Wilcoxon signed-rank test was used for the low-ranked item subanalysis in the HAM-D_17_ in PD. Furthermore, the Mann-Whitney's *U* test was used to compare items in the PD group with those in each control group.

#### 2.4.3. Results

**(A) Prevalence Rates of Depression and Apathy**

Table 4 shows the depression and apathy prevalence rates. The depression prevalence rate in the PD group was 19.6%, and this rate was almost equal to that in the stroke group. The apathy prevalence rate in the PD group was as high as 50%, and this rate was much higher than those in the control groups. Moreover, 88.9% of PD patients who were diagnosed with depression also had apathy, with a complication rate remarkably higher than those of the other groups.

**(B) Subanalysis of Nine Low-Ranked Items of the HAM-D_17_ in the PD Group**

**Figure 1:**
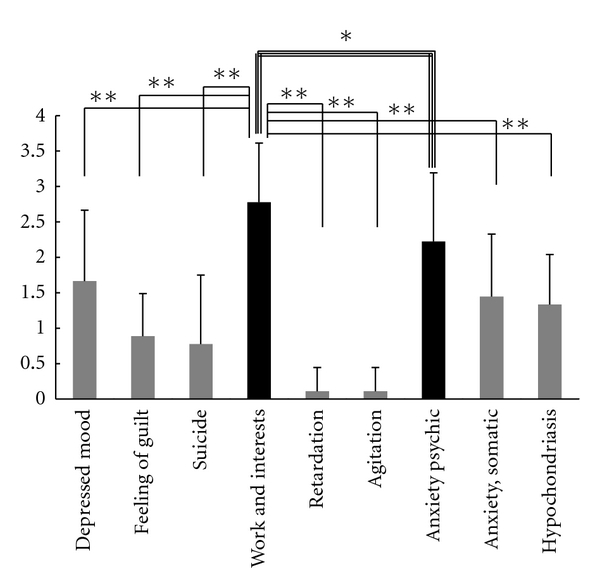
Result B of examination 2-2. Character of depression in PD. n.s. *P* < .05 (Wilcoxon signed-rank test).

Table 5 shows the average score ± SD of each of the nine low-ranked items of the HAM-D_17_ in the PD group and the three control groups.  Figure 1 shows a graph of the average PD group scores. Among the nine items, “work and interests” obtained the highest average score, followed by “anxiety, psychic.” No significant difference was observed in the average score between these two items in the signed-rank test. However, the average scores of the other seven items, such as “depressed mood,” were significantly lower than that of “work and interests.”

**(C) Comparison of the PD Group with the Control Groups (Stroke, MOH, and Endogenous Depression)**

The average scores of the HAM-D_17_ items “depressed mood,” “retardation,” and “agitation” were significantly lower in the PD group than in the endogenous depression group (Table 5). Particularly, the average score of the item “depressed mood” was lower in the PD group than inthree control groups. In contrast, the average scores of the item “work and interests” and items that reflected anxiety in the PD group were similar to those in the endogenous depression group.

#### 2.4.4. Discussion

The results in Section 2.4.3 (A) show that the prevalence rate of apathy is much higher in the PD group than in the control groups. This finding supports the results reported by Kirsch-Darrow et al. [21], who described that apathy is the core feature of PD. The complication rate of depression and apathy described in Section 2.4.3 (A) and the results described in Section 2.4.3 (B) and (C) support the hypothesis that in PD patients apathy is generally and frequently observed, even in the state of depression. Section 2.4.3 (C) shows that no significant difference was observed in the average score of “work and interests” between the PD group and endogenous depression group, and Section 2.4.3 (B) describes that a significant difference was observed in the average score between “work and interests” and “depressed mood,” “feeling of guilt,” and “suicide.” These results indicate that in PD patients with depression the frequencies of decreased willingness and loss of pleasure are higher, and the frequencies of feeling of guilt, self-accusation, and feeling of loss are lower, and also the rate of suicide is lower than in patients with “endogenous depression.” [13] 

Similar to depression and apathy, PD is typically accompanied by anxiety disorders, which are observed in approximately 40% of PD patients [3], although this rate differs among various reports. Anxiety disorders are caused by decreased serotonin following dorsal raphe nucleus denaturation due to PD progression, or by overactivity of the noradrenaline system caused by the disinhibition of the dopamine nerve to the locus coeruleus. In this study, we did not diagnose anxiety disorders in accordance with the DSM-IV, and therefore, the detailed mechanism of anxiety disorders is unknown. However, the results related to anxiety in (C) suggest that similar to depression in PD, anxiety disorders can also become a complication in PD.

## 3. Definition and Morbid State of Anhedonia

### 3.1. Apathy and Anhedonia

Similar to apathy, anhedonia [4] has recently attracted attention as an NMS in PD. Apathy is mainly characterized by loss of primary motivation, loss of interest in the environment, and affective dullness. Anhedonia is the state in which the patient cannot derive essential pleasures from behaviors and activities that were joyfully performed in the past. In other words, anhedonia is a morbid state in which the patient's sensitivity to pleasure has decreased. Similar to apathy, anhedonia is not defined in the DSM-IV. If anhedonia is forcibly expressed, it is the state in which only “loss of pleasure” is manifest, but not “loss of interest” as shown in Table 1. Anhedonia in PD can be explained as follows: (a) disturbance of the dopaminergic circuit, which projects dopamine from the mesolimbic system to the frontal cortex, affecting the reward system in the neurodegenerative process; (b) decreased motivation; (c) decreased willingness, decreased spontaneity, loss of sociality, and loss of interest in joyful stimuli (sexual acts, eating, smoking, drinking, etc.) [4, 26]. 

As mentioned above, anhedonia has nearly the same meaning as “loss of pleasure” in the diagnostic criteria of major depression in the DSM-IV. Among symptoms that have been reported as anhedonia in PD, decreased willingness and emotional disorders are also observed in apathy. Moreover, the mechanism, which is assumed to be a reward-system disturbance in the dopamine system, is common to apathy and anhedonia. Lemke et al. [4] conducted an open study to evaluate the effect of pramipexole and reported that anhedonia was observed in 45.7% of the 626 PD patients involved in the study. This value is similar to the apathy prevalence rate (approximately 40%) [3].

The relationship between apathy and anhedonia in PD has not been explained in detail. Pluck and Brown[27] investigated apathy in 45 PD patients and reported that in cases where apathy was clearly observed, anhedonia was also significantly present. Moreover, no relationship between apathy and depression was observed. Therefore, apathy and anhedonia are considered to have similar mechanisms and morbid states.

To evaluate anhedonia, the Snaith-Hamilton Pleasure Scale (SHAPS), created by Snaith et al., can be used [28]. SHAPS uses a self-recording system and consists of 14 question items. Since these items are scarcely affected by motor functions, SHAPS can be used to evaluate anhedonia in PD patients. Unlike AS, SHAPS cannot diagnose anhedonia on a stand-alone basis (anhedonia is “suspected” when the score is 3 or higher). However, SHAPS is useful in determining the effects of therapy. The study conducted by Lemke et al. employed SHAPS.

### 3.2. Examination Results Obtained in Our Institute—Part 2: Apathy and Anhedonia

The relationship between the morbid states of apathy and anhedonia in PD was examined.

#### 3.2.1. Subjects

In this examination 50 PD patients were used as subjects (Tables 5 and 6).

#### 3.2.2. Methods

Using diagnostic criteria defined in the DSM-IV we diagnosed depression (major depression, minor depression, or dysthymia). Moreover, using the AS Japanese version [23] and SHAPS, apathy and “suspected” anhedonia were diagnosed and quantified. Prevalence rates of depression, apathy, and anhedonia were calculated, and their complication rates were investigated. The correlation between AS and SHAPS was evaluated using the Spearman's correlation coefficient by rank test.

#### 3.2.3. Results

**(A) Prevalence Rate**

**Figure 2:**
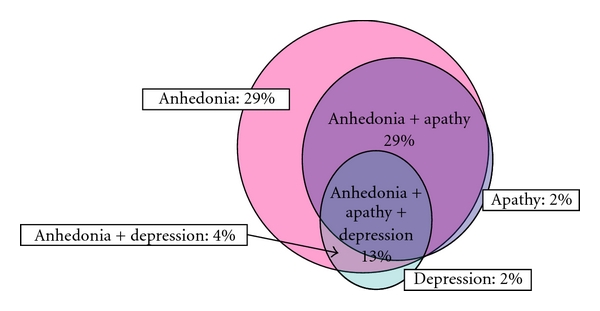
Result A of examination 2. Prevalence of apathy, anhedonia, and depression in PD.

Prevalence rates of depression, apathy (AS ≥ 16), and “suspected anhedonia” (SHAPS ≥ 3) were 18%, 44%, and 74%, respectively. As shown in Figure 2, nearly all cases diagnosed with depression were complicated with apathy and anhedonia, and nearly all cases diagnosed with apathy were complicated with anhedonia.

**(B) Relationship between Apathy and Anhedonia**

**Figure 3:**
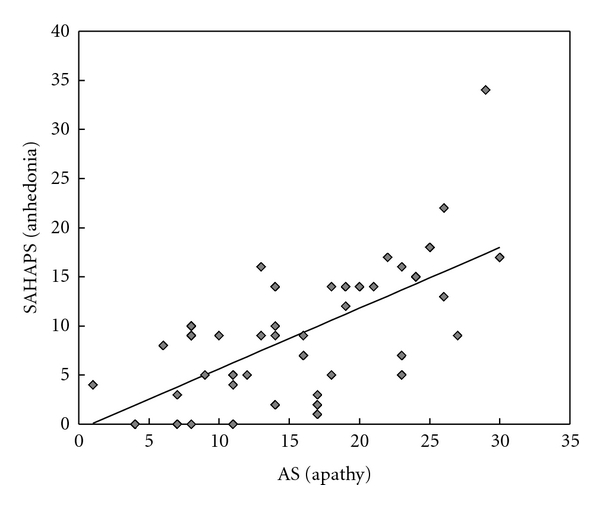
Result B of examination 2. Correlation between apathy score and SHAPS.

Figure  3 shows the correlation between AS (apathy) and SHAPS (anhedonia). As shown in this figure, a strong correlation (*r* = 0.64 and *P* = .00000065) was obtained between AS and SHAPS.

#### 3.2.4. Discussion

**Figure 4:**
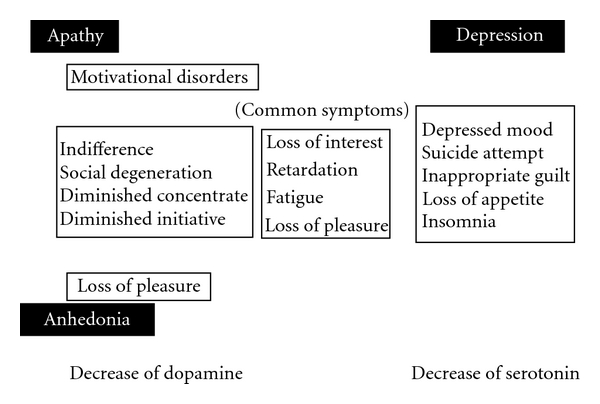
Relationship of apathy, anhedonia, and depression.

The prevalence rates of depression and apathy are similar to those in the examination results from part 1. The prevalence rate of “suspected anhedonia” was significantly higher than that in the report by Lemke et al. Since SHAPS was used in the present study and had been translated into Japanese by the author of this paper and since the validity of SHAPS in Japanese has not been established, the prevalence rate of “suspected anhedonia” may be overestimated. However, nearly all patients diagnosed with “suspected” anhedonia were also diagnosed with apathy. Moreover, a strong correlation was obtained between AS and SHAPS, as described in (B). Therefore, apathy and anhedonia share a common mechanism (dopamine exhaustion disturbances of the reward system). Thus, apathy and anhedonia are core features of mood disorders in PD and are independent of depression in Japanese patients.  Figure 4 shows an approximate relationship between apathy, anhedonia, and depression in PD.

## 4. Therapy for Apathy and Anhedonia (Pharmacotherapy)

At present, there are no proven medicines for apathy and anhedonia in PD, and the suitability of PD therapy for treating apathy and anhedonia needs to be evaluated. When PD is strongly suspected to be caused by dopamine deficiency (e.g., therapy has been extremely insufficient, or apathy or anhedonia appears only in the off stage), dopaminergic therapy may be effective. In particular, pramipexole, a dopamine agonist, is reported to improve the symptoms of depression and decreased willingness. However, such results were mainly obtained in open studies, and the therapeutic methods differ according to the opinions of specialists. Additional dopaminergic therapy in patients who show sufficient control over motor symptoms may increase the risk of inducing adverse effects, such as lower limb edema, daytime drowsiness, valvular disease of the heart, mental symptoms, and dyskinesia. Therefore, the so-called “made to order therapy” in consideration of the patient's background is essential. 

### 4.1. Dopaminergic Therapy

Apathy or anhedonia in PD exhibits similar symptoms to subcortical dementia, in which the motivational disorder caused by a disturbance in the dopamine projection system of the mesencephalon frontal cortex plays a central role [1]. If true, dopaminergic therapy should be the first choice to treat apathy and anhedonia in PD patients. Among dopamine agonists, pramipexole, pergolide, and ropinirole are reported to be effective in treating decreased willingness and depression in many cases [29]. Particularly, pramipexole has a stimulatory action on D2 receptors in the nigrostriatal dopamine system related to the motor system and D3 receptors. D3 receptors are widely distributed in the mesolimbic pathway (from the ventral mesencephalon to the nucleus accumbens) and the amygdaloid body in the dopamine projection system in the mesencephalon frontal cortex. The D3 stimulatory action of pramipexole in these emotion-related sites is presumed to improve symptoms of apathy and anhedonia [30]. There have been several large-scale open studies on anhedonia.Reichmannet al. [26] administered pramipexole to 135 anhedonia patients, and Lemake et al. [4] administered pramipexole to 286 PD patients with anhedonia. In these patients, anhedonia significantly improved in addition to motor symptoms. In 2010,Bxaroneet al. [31] reported that pramipexole is effective for treating depression in PD. Because a randomized double-blind method was adopted, the confidence level of the obtained results was high.

Several reports have described that selegiline, a monoamine oxidase B (MAO-B) inhibitor, is effective for the treatment of decreased willingness and depressed mood in PD.Tom and Cummings[32] recommended selegiline as the first choice to treat depression in PD patients not exhibiting suicidality. The primary effects of selegiline are inhibition of MAO-B and efficient use of intracranial dopamine. Moreover, selegiline is known to reinforce the intracranial phenylethylamine (PEA) activity [33]. PEA promotes serotonin release to improve symptoms of decreased willingness and depressed mood in PD. Therefore, PEA is particularly effective for apathy and anhedonia complicated with depression. Unlike dopamine agonists that are typically used alone, selegiline can be used in combination with other medicines, that is, the so-called “add-on use” is possible. Therefore, selegiline is a simple and convenient drug. However, since selegiline used in combination with antidepressants (which are described below) is contraindicated, care must be taken when administering selegiline.

Since amantadine hydrochloride induces dopamine secretion and exhibits a catecholamine stimulatory action, this medicine is useful to treat PD. Because of its mechanism, the effect of amantadine hydrochloride on apathy is expected. Amantadine hydrochloride is used to treat apathy in PD as a supplemental therapy [34].

### 4.2. Antidepressants

Few reports exist on the effects of various antidepressants on apathy in PD (without depression). Selective serotonin reuptake inhibitors (SSRIs), serotonin noradrenaline reuptake inhibitors (SNRIs), tricyclic antidepressants (TCAs), and tetracyclic antidepressants were reported to be effective therapies for major depression occurring along with PD. In particular, the effect of the TCA nortriptyline [35] was reported to be effective in a randomized controlled trial with a placebo group. The positive effects of the SSRIs sertraline [36] and paroxetine [37] on depression in PD patients were reported in open studies based on more than 100 cases. The American Academy of Neurology (ANN) recommends amitriptyline for the treatment of depression in PD [38]. These results were obtained on the basis of depression, not apathy or anhedonia alone. Generally, antidepressants are not effective for the treatment of depression in which remarkable decrease in willingness is observed. On rare occasions, antidepressants worsened the decreased willingness [39]. Thus, antidepressants should not be imprudently used for cases where apathy or anhedonia is independently observed. Maruyama[40] administered milnacipran, an SNRI, to eight PD patients with depression and reported that it was effective in treating depression distinctive to PD, including cases where significant apathy or anhedonia was observed. Moreover, milnacipran administration caused total remission in cases of depression with PD for which an SSRI was not effective [41]. According to the monoamine hypothesis, SNRIs, which have significant noradrenaline-inducing activity, are effective for depression in which apathy or decreased willingness is remarkably significant [42]. Since the effects of SNRIs on depression and decreased willingness are expected in cases for which pramipexole is not sufficiently effective, the validity of SNRIs must be established.

## 5. Conclusion

Together with depression, apathy and anhedonia often develop in patients with PD. Since the morbid states of apathy and anhedonia are complicated, these symptoms are often difficult to diagnose. Several therapeutic methods for apathy and anhedonia are considered effective. However, the validity of these methods has not been established. Similar to depression, apathy and anhedonia clearly affect the quality of life of patients and their families. Therefore, accurate diagnoses of morbid states in the early stage of the disease and corresponding appropriate treatments are more important than ever.

## Figures and Tables

**Table 1:** DSM-IV criteria of major depression.

	Items
(1)	Depressed mood
(2)	Loss of interest or loss of pleasure
(3)	Significant weight loss or loss of appetite
(4)	Insomnia or hypersomnia
(5)	Psychomotor agitation or retardation
(6)	Fatigue or loss of energy
(7)	Feelings of worthlessness or excessive or inappropriate guilt
(8)	Diminished ability to think or concentrate, or indecisiveness
(9)	Suicide attempt or a specific plan for committing suicide

**Table 2:** Values of the prevalence rate of depression occurring along with PD.

	Author	*n*	Depressed state	Major depression	Dysthymia
1986	Santamaria	34	32.3	2.9	29.4
1988	Mayeux	339	47		
1990	Brown	40	25		
2000	Cubo	88	7.3	7.3	
2000	Goladi	172	33		
2003	Krishnan	126	12.7		
1995	Tison	60	32.7		
2001	Happe	56	76.4		
2001	Shulman	99	36		
2002	Happe	116	37.1		
2002	Marinus	177	38.4		
2002	Shilman	101	44		
2003	Roji	353	56.9		
2005	Hely	52	53.6		
2005	Holroyd	100	15		
2005	Prado	60	38.3		
2006	Kirsch-Darrow	80	26.3		
2006	Weintraub	130	36.2		

**Table 3:** Materials of examination 1. mRS: modified Ranking Scale.

	*n*	Male	Female	Mean age ± SD (year)	Other conditions
PD	46	16	30	64.6 ± 10.5	Duration from onset 60 ± 48.6 SD (month) Hoehn and Yahr scale I: 1 II: 5 III: 29 IV: 11 treated; 32 not treated: 14
Stroke	54	19	35	66.5 ± 10.6	Duration from onset:17.9 ± 4.9 SD (day) mRS Grade 0 : 5 Grade 1 : 14 Grade 2 : 15 Grade 3 : 12 Grade 4 : 8
MOH	20	7	13	41.6 ± 15.7	All treated
Endogenous depression	10	4	6	52.0 ± 22.6	All not treated

**Table 4:** Result A of examination 1. Prevalence of mood disorders in PD and control groups.

	*n*	Depression (major depression)	Apathy	Apathy/depression
PD	46	19.6% (6.5%)	50.0%	88.9%
Stroke	54	18.5% (1.8%)	35.2%	50.0%
MOH	20	65.0% (20.0%)	35.0%	46.0%

**Table 5:** Result B of examination 2-1. Differences between PD and control groups in HAM-D_17_ subitems. *P* < .05 Mann-Whitney U test.

	Items	PD	Stroke	MOH	Endogenous depression
1	Depressed mood	1.44 ± 1.13*	1.78 ± 1.09*	2.08 ± 1.04	2.78 ± 0.67
2	Feeling guilt	0.78 ± 0.67	0.56 ± 0.73	1.31 ± 1.11	1.11 ± 0.93
3	Suicide	0.44 ± 0.53	0.44 ± 0.73	0.92 ± 1.04	1.56 ± 1.51
7	Work and interests	2.56 ± 1.01	1.44 ± 0.73*	2.23 ± 0.80	2.44 ± 1.01
8	Retardation	0.11 ± 0.33*	0.56 ± 0.73	0.31 ± 0.50*	0.89 ± 0.78
9	Agitation	0.00*	0.33 ± 0.50	0.31 ± 0.50*	0.44 ± 0.53
10	Anxiety, psychic	2.22 ± 0.97	1.67 ± 1.12	2.31 ± 1.20	2.44 ± 0.88
11	Anxiety, somatic	1.56 ± 1.01	1.67 ± 0.87	2.46 ± 0.90	1.44 ± 0.53
15	Hypochondriasis	1.22 ± 0.67	1.44 ± 0.88	1.15 ± 1.14	1.56 ± 0.88

**Table 6:** Materials of examination 2.

Sex	Male: 27	Female: 23
Mean age (year)	67.8 ± 11.7 (SD)	
Duration from onset (year)	8.1 ± 5.4 (SD)	
Hoehn-Yahr scale	II: 8, III: 31, IV: 11	
Treatment	Treated: 46	Not treated: 4
